# ‘Moko’ drums and gongs, ritual musical instruments and local currency from Alor Island, Southeast Indonesia: A comprehensive and verified lexical data set

**DOI:** 10.1016/j.dib.2024.110354

**Published:** 2024-03-21

**Authors:** Shiyue Wu, Francesco Perono Cacciafoco

**Affiliations:** Xi'an Jiaotong-Liverpool University (XJTLU), School of Humanities and Social Sciences (HSS), Department of Applied Linguistics (LNG), 8 Chongwen Road, Suzhou (Jiangsu), 215123, China

**Keywords:** ‘Moko’ kettle-drums, Alor-Pantar Archipelago, Papuan Indigenous Cultures, Abui, Sawila, and Kula, Language Documentation

## Abstract

This paper provides comprehensive and systematized lists of names of ‘moko’ drums from Alor Island, in Southeast Indonesia. ‘Moko’ drums are unique cultural objects from the Alor-Pantar Archipelago and, besides their ancient function of ritual instruments used mainly for religious purposes and in public events by the indigenous peoples of the islands, they represented and still are considered a very valuable local ‘currency’ for trade and for specific social interactions rooted in aboriginal culture, like bride price negotiations. Despite the fact that they are extremely popular and widespread among Papuan peoples in Alor and Pantar, the origins of these drums are still relatively obscure. The native speakers, indeed, cannot explain the name ‘moko’ in itself, at the etymological and semantic level, and, despite the fact that they agree upon non-local origins of the instruments, they do not know where the instruments themselves were produced and from where they came to the islands. Our paper provides the readers with comprehensive lists that systematically collect the names of the drums, with the related glosses and basic additional information, from three representative Papuan languages of Alor Island, namely Abui, Sawila, and Kula. Configured as potentially indispensable tools to develop further research, these lists enhance our knowledge and understanding of the culture of the ‘moko’ drums in the Alor-Pantar Archipelago, at the linguistic (etymology of the names), anthropological (social value of the drums), and archeological (typology and provenance of the instruments) levels. This cataloguing operation is also part of the effort of documentation of the languages and cultures, still scarcely documented and definitely endangered, of the native peoples of the Alor-Pantar Archipelago.

Specifications Table

**Table:** 

Subject	Language Documentation / Field Linguistics
Specific subject area	Documentation of specific sets of culturally relevant lexical items designating ritual drums and gongs from indigenous undocumented and endangered languages in Southeast Indonesia
Data format	Raw data in .xlsx format
Type of data	Set of tables systematically collecting and categorizing the names of the drums and their glosses / translations language-by-language, with a set of pictures aimed at a direct visualization of the most relevant items in the lists
Data collection	Active Language Documentation fieldwork, performed and finalized between October and December 2023, with Abui, Sawila, and Kula native speakers and consultants in and from Alor Island, Alor-Pantar Archipelago, Southeast Indonesia, Timor area; intensive interviews for data collection; transcription of the results and findings; validation of collected data with the help of the Curators of the local Museum (Kalabahi, Alor)
Data source location	Alor Island, Alor-Pantar Archipelago, Southeast Indonesia, Timor area, Abui, Sawila, and Kula territories (Central and Eastern Alor)
Data accessibility	Our data set is uploaded on Mendeley Data. Repository name: Name List of ‘Moko’ Drums in Abui, Sawila, and Kula Data identification number: doi:10.17632/zwkbykdbrv.1 Direct URL to data: https://data.mendeley.com/datasets/zwkbykdbrv/1

## 1 Value of the Data

• The data is a comprehensive list of ‘moko’ drums’ names in three representative Papuan languages from Alor Island, i.e., Abui, Sawila, and Kula, evidence of cultural continuity and contact among the different local ethnic groups;
• the data is an indispensable source for Language Documentarists, Cultural Anthropologists, and Archeologists, to further the investigations on the origins of the ‘moko’ drums at the linguistic level and in the contexts of social customs and religion among the local aboriginal communities, as well as on the material culture of the peoples of Alor Island;
• being structured as systematic lists, this data can contribute to the expansion of the debate on the location of the original places of production of the ‘moko’ drums, sometimes possibly foreshadowed by the names of the drums themselves – this would significantly enhance our knowledge of trade routes and cultural contexts in the related areas of Southeast Indonesia;
• the data can offer a solid foundation to build up a new attempt of etymological reconstruction for the word ‘moko’ in itself, which, despite the fact of being widespread in Southeast Indonesia, has, apparently, no linguistic explanation;
• the lists add up, in the format of ‘first-hand’ data, to the research so far produced on ritual and trading drums in Alor and to the documentary effort of safeguard and preservation of the cultures and languages of the Papuan peoples of Alor Island;
• the data will allow to apply a comparative approach to the assessment and analysis of the drum names, which is methodologically indispensable, both at the linguistic level and at the typological (material culture) level.

## 2 Background

‘Moko’ is the widespread and generalized term for bronze kettle-drums commonly findable across the entirety of the islands of Alor and Pantar (and neighboring areas). This denomination could have originated from Alor and/or Pantar, possibly emanating from a local *lingua franca*, ‘Alor Malay’, already used across the Alor-Pantar Archipelago since the 14^th^ century, after traders settled in the place [1]. As briefly mentioned in the abstract, these bronze drums are greatly valued by the different local ethnic groups among the Alor peoples and are essential negotiation tools in bride price practices. Indeed, they were and are used by the native populations not only as ritual musical instruments and symbolic objects, but also as a sort of local currency and prestigious material elements in epichoric trade [2], [3], [4], [5]. Moreover, the drums were utilized in public religious ceremonies (still continued in local folklore and celebratory events) and represent a valuable and unique form of currency in bride price negotiations, being also prestigious heirlooms in and for each community [2,^3^,6].

A magnificent collection of ‘Moko’ drums is hosted at the local Kalabahi Museum in Alor Regency, which provides the visitors with exhaustive lists of names in three characteristic languages from the Alor-Pantar Archipelago, i.e., Abui and Kabola, from (Central and Northern) Alor, and Pantar, from Pantar. However, all different ethnic groups in Alor and Pantar (and surrounding areas) have their own onomastic variants, for the different drums, with specific ritual and trading characterizations.

Our data set focuses on Abui, Sawila, and Kula, because the three contexts are very indicative of the patterns of similarities and individual divergences in the usage and perception of these culturally significant items by the different peoples from the area. Indeed, Abui, Sawila, and Kula are Papuan languages spoken in the Alor-Pantar Archipelago in Southeast Indonesia, specifically in the Central and Eastern parts of Alor Island. The names of the languages also represent the related ethnonyms (with some variants). The three languages are related (despite Sawila and Kula show a higher degree of divergence from Abui and relatedness to each other) and are still partly undocumented (especially Sawila and Kula). In the last twenty years, a relevant effort was produced, by some Language Documentation teams, to safeguard them [7], [8], [9], [10], [11], [12], [13]. In particular, recent research on Abui culture and Toponymy [14], [15] contributes to the preservation of collective memories and legends [16], [17] and linguistic and social elements among local inhabitants. However, due to the limited number of speakers still fluent in their native languages in the archipelago and the widespread use of *Bahasa Indonesia* as a common language, the Alor-Pantar languages should, unfortunately, still be considered endangered.

Many questions inherent in the drums have not been answered yet, notwithstanding a respectable amount of research on their general contexts. For example, the geographical and cultural place or places of origin of the drums themselves have not been located yet [18]. The same name ‘moko’, which, ideally, indicates all the different typologies and categories of these instruments, has, so far, eluded the attempts to reconstruct its etymology, and its original linguistic context (and meaning) is still obscure. The paradox, indeed, lies in the fact that the term is commonly used, in the everyday speech, by all the Alor-Pantar speakers, but, apparently, nobody, among them, can gloss it or guess its possible origins. This, with the sometimes confused or overlapping beliefs of the indigenous peoples, directs the reconstruction efforts towards the postulation of a non-local provenance of the drums and the related ‘all-embracing’ word indicating them. Hence, the documentation of specific sets and sub-sets of lexical items and specialized vocabulary from these languages is essential to enhance our chances to effectively preserve them. Our paper, therefore, provides the current panorama of studies on the three languages with a specific tri-lingual data set which can be analyzed not only at the linguistic level, but also inherently in the Cultural Anthropology and material culture (and Archeology) of these indigenous peoples from Alor.

At the introductory level, it is necessary to spend some words on the distinction between ‘bronzes’ (bronze drums) and ‘gongs’ in Alor's culture. The paper, indeed, presents additional lists of names for several categories of another percussion musical instrument widely used in the Alor-Pantar Archipelago, the ‘gong’. The ‘gongs’ are typologically and culturally linked to the standard bronze drums and can be assimilated to them, but, in their specificity, they are round, flat metal discs typically played by hitting them with a mallet. They vary in sizes, ranging from small to large. Like the drums, the gongs seem to have a non-local origin and are used for ritual music and trade in and to Alor (and neighboring areas). Over time, they were utilized by local peoples not only for musical performances, but also for bride price negotiations and commercial exchanges [2], and this makes them assimilated to the bronze drums also at the functional level.

## 3 Data Description

**The data set includes:**

• the original names of the different types of ‘moko’ drums from three ethnic groups in Alor Island, i.e., Abui, Sawila, and Kula, highly representative of the mutual diversity and consistency of the culture in the island (Abui from Central Alor and Sawila and Kula from Eastern Alor);
• the related glosses, name-by-name, with very synthetic notes (where available) on the possible etymology and lexical interpretation;
• a subdivision and grouping of the instruments, by typological categories, based on information collected from the local native speakers and direct observation of the related material culture;
• the different linguistic layers of the ‘naming’ of the drums in the three respective languages;
• a set of pictures reproducing a small selection of drums according to their cultural significance.

### 3.1 Bronze drum (‘Moko’ drum) and gong name lists

Note: ‘moko’ is the *lingua franca* word used by local peoples, in Alor Island, to generically indicate the ritual and trading bronze drums. Each ethnic group employs also different (local) words (some of them are quite ‘cryptic’, in their meaning, and ‘obscure’, in their etymology) to refer to the same drums. In our data, the label ‘bronze drum’ is equivalent to ‘moko drum’ (Table 1, Table 2, Table 3, Table 4).

**Table 1:** Names of the ‘moko’ drums in Sawila.

Bronze Names (categorized by value, and from smaller to bigger)	Gloss	Gong Names	Gloss
*piku*	lit. ‘small’	*tatabung (tata-bung)*	lit. ‘lay down’; ‘put down (onomatopoeia = '*bung*')’
*piku makeser*	lit. ‘*Makasar*-small’	*tabongala*	
*jawa piku*	lit. ‘*Jawa*-small’	*tipawaline (tipa-waline)*	lit. ‘new-drop’ (‘one down’)
*kaluma (kalu-ma)*	lit. ‘net-inside’	*empolo*	lit. ‘complete gong’
*maruko ulmale*	lit. ‘rotten (ancient)-one down’	*lonamanakalala (lona-manakalala)*	lit. ‘smooth’; ‘slippery-village gong’
*maruko makeser*	lit. ‘rotten (ancient)-*Makasar*’	*serengmanakalala (sereng-manakalala)*	lit. ‘lower-village gong’
*makasar lotarmara (lotar-mara)*	lit. ‘*Makasar*-rattan-exist’	*manakalala (mana-kalala)* *managung*	lit. ‘village-gong’ - ‘*kalala*’ is the dialectal form from Eastern Alor's coastal area for the word ‘*gung*’ in Sawila
*wandaulmale*	lit. ‘*wanda*-one down’	*kung giya*	lit. ‘gong-its mother’
*wanda*			
*makasar*	lit. ‘*Makasar*’		
*jawa makasar*	lit. ‘*Jawa*-*Makasar*’		
*jawa*	lit. ‘*Jawa*’		
*yaulmale (ya-ulmale)*	lit. ‘down-lower than-higher’		
*ulmale (ul-male)*	lit. ‘lower than-higher’		
*malesaso*	lit. ‘higher-bronze drum’

**Table 2:** Names of the ‘moko’ drums in Kula.

Bronze Names (categorized by value, and from smaller to bigger)	Gloss	Gong Names	Gloss
*saso piku*	lit. ‘bronze drum-small’	*kingkung (king-kung)*	lit. ‘*king kung*’ (onomatopoeia = ‘*gong*’)
*saso makesa (lawa)*	lit. ‘bronze drum-*Makasar*’ (‘rotten’; ‘ancient’)	*tatabung*	
*saso jawa*	lit. ‘bronze drum-*Jawa*’	*kung gia*	lit. ‘*gong*-its mother’
*saso*	lit. ‘bronze drum’	*kung gipa*	lit. ‘*gong*-its father’
*saso waikik*	lit. ‘bronze drum-*Makasar*-candlenut’		
*saso pilawaka*	lit. ‘bronze drum-stamped-moon’		
*saso waikik gigis*	lit. ‘bronze drum-candlenut-fruit’		
*saso waikik gilaka*	lit. ‘bronze drum-candlenut-flower’		
*saso gulmalei*	lit. ‘bronze drum-highest’

**Table 3:** Names of the ‘moko’ drums in Abui.

Bronze Names (categorized by value, and from smaller to bigger)	Gloss	Gong Names	Gloss
*piku*	lit. ‘small’	*keeng-keeng*	lit. ‘*keeng-keeng*’ (onomatopoeia)
*faat tafaa*	lit. ‘corn-drum’	*fuoqukung (fuoqu kung)*	lit. ‘ankle-gong’
*lasing tafaa*	lit. ‘bracelet-drum’	*raai*	lit. ‘eucalyptus tree’
*hawaa taq(k)a*	lit. ‘its mouth-empty’	*hora-hora*	‘*hora*’ is a ‘mimetic’ word, used to express the mood of fear when one sees the flames of a fire
*teeng sama (tuang sama)*	lit. ‘*teeng sama’* (onomatopoeia + ‘*sama*’)		
*tama mia*	lit. ‘middle drum’		
*fe hawa*	lit. ‘pig-mouth’		
*aimaala (ai-mala)*	lit. ‘*Makasara-e-hei*’		
*maneeng (ma-neeng) maak*	lit. ‘female youth’, ‘man-belt’		
*ia kasing*	lit. ‘moon-half’		
*fiyaai futal*	lit. ‘candlenut-flower’		
*manei taka*			
*namang wea*	lit. ‘clothes-bloody’		
*jawa (yaawa)*	lit. ‘*Jawa*’		
*yawa hawei*	lit. ‘*Jawa*-with ears’		
*bileeqwea (bileeq-wea)*	lit. ‘lizard-blood’		
*kolmalei (kol-malei)*	lit. ‘women name-*maleei* (‘*maleei*’ = name of a liana)’		
*itkira (it-kira)*	lit. ‘lay down-hard’

**Table 4:** Some images of ‘moko’ drums from the Kula ethnic group with Abui lexical correspondences.

The macro-categories of ‘bronzes’ and ‘gongs’ show a multi-layered degree of distinction and differentiation, based not only on the perceived value, size, and the material features of the instruments, but also on their possible ‘ideal’ provenance (*Jawa* vs *Makasar* and/or *Jawa* and *Makasar*, transcribed here by following the consultants’ spelling), according to the beliefs of the speakers, which, almost paradoxically, derive from the existing names. The geographical ‘coordinates’ of the original lands of production of these instruments relate to the initial stages of the history of trade between Alor and Sulawesi [19], but their locations have been lost over time, with the memories of the local peoples. The places of origins of the drums, indeed, have not been confirmed at the current stage of the related research yet, and the local populations are quite confused, when asked about the possible original territories of production of the instruments. What they seem to agree upon, however, is that the drums are not crafted locally, i.e., in the Alor-Pantar Archipelago.

In the cases where a gloss is not included in the tables our native speakers, and even the existing cataloguing documentation, are unsure about or incomplete inherently in the possible meanings of the names. We have decided, therefore, rather than attempting etymological reconstructions which cannot be proven, at this stage, to leave the related ‘pigeon-holes’ empty, to provide the scholars interested in working on these names with a consistent list without any additional interpretation bias.

‘Bronzes’ and ‘gongs’, despite the fact that their origins look ‘obscure’ and undocumented, are accurately differentiated, catalogued, and valued by the indigenous communities – a sort of multi-ethnic local council, in fact, gathers periodically to establish and officialize the different categories and levels of social prestige and rarity of every single instrument.

The onomastic and onomasiological stratification of the names and the typological classification of ‘bronzes’ and ‘gongs’ indicate their high value and cultural pervasiveness among the local aboriginal societies (the identitarian value in itself is intricately and intrinsically linked to the affective and emotional onomastic perception among local peoples, in a parallel with, *mutatis mutandis*, a study on the effects of social naming and renaming [20]). This appears to be in contradiction, somehow, with the speakers’ lack of knowledge and/or memory regarding the origins of these ritual and trading objects, whose places of production, as mentioned, have not been confirmed nor located yet.

## 4 Experimental Design, Materials, and Methods

Our data collection was developed according to a classic Language Documentation method based on fieldwork and interviews with local native speakers in Alor Island. Specifically, we were able to rely on the constant collaboration of three indigenous consultants, Mr Benidiktus Delpada (born in Takalelang on September the 19^th^, 1984 – a multilingual local linguist and researcher and Abui native speaker who constantly cooperates with the Universitas Tribuana Kalabahi – Tribuana University of Kalabahi, Alor), who coordinated the consultations with Mr Pak Otniel (born in Kaipera on October the 24^th^, 1965 – a teacher of secondary school and Kula native speaker) and Mr Pak Aris (born on August the 24^th^, 1957 – a former teacher and Sawila native speaker). Pak Otniel lives in Kaipera, Desa Tanglapui, Kecamatan Alor – Timur, while Pak Aris lives in Kelurahan Nusa Kenari. Both native speakers are elders, in their communities, and master their respective indigenous languages. The three consultants double-checked and verified the collected data with their respective communities. While our Language Documentation work with these indigenous native speakers is conducted on a larger and exhaustive scale (involving grammar, lexicon, oral traditions, genealogies, and place names of and from their languages), for this specific task inherent in the ‘moko’ drums we were able also to rely on the valuable help of Ms Ibu Yanti, a Kabola speaker from the Kalabahi Museum in Alor Regency, who provided us with specific details and information on the instruments which appear in our list.

All interviews and research work, as well as the direct interaction with local people, have been conducted according to the highest standards of ethics in Field Linguistics. All the consultants were provided with an exhaustive informed consent form to read and sign, and extensive information and details on our research, methodologies, ethical implications, data storage, and personal data treatment were explained to them before our study began, while the works were in progress, and in the end, when the collected data was already stored and processed. Each consultant was duly and timely compensated for the work developed, and we also proceeded to micro-donations aimed at the local communities through our collaborators, with the goal of language preservation.

Being the aim of this paper to provide Language Documentarists and Cultural Anthropologists with a linguistically well-organized, but essentially ‘raw’ set of ‘first-hand’ data inherent in the ‘moko’ drums, we kept the comparative (and contrastive) analysis to a minimum and we focused on the development and rationalization of the data set. The hope is to offer a significant source for historical and comparative studies, while we work on the drum names in the broader context of our research on Alor-Pantar Lexicology and Documentary Lexicography.

As mentioned, our categorization of the relevant terms and the subsequent cataloguing operation are, indeed, aimed at producing a comprehensive list in the three different Papuan languages from Alor, useful not only to enhance and develop more in-depth linguistic, anthropological, and archeological research on the drums themselves and their cultural valency and significance, but also to produce comparative studies, at the onomastic / onomasiological level and at the level of typology – in the context of material culture –, on these unique musical instruments.

The goal is to help scholars from around the world to further our understanding of the languages and social dynamics of the indigenous Papuan peoples living in Alor Island and in the Alor-Pantar Archipelago.

Additionally, our paper would like to be a contribution to the Language Documentation efforts aimed at preserving and safeguarding the languages spoken in Alor and the related oral traditions and intangible heritage of the local populations, among which the ‘moko’ drums are of considerable importance both at the level of cultural identity and in the context of social conventions and interactions.

## Limitations

Despite the fact that our data set is, to date, the most comprehensive list of ‘moko’ drum names from Alor, a natural limitation of our research is represented by the issue that it is not possible, due to limits of manpower and established relationships with local speakers in the communities of the islands, to collect and document in a timely manner analogous lexical lists from other ethnic groups in the Alor-Pantar Archipelago. We feel, therefore, that the publication of our data set can be an important starting point in this documentary, etymological, and archeological research, aimed, as mentioned, at encouraging other scholars to complete the related lexical lists through active fieldwork in the islands. At the personal level, we are planning to eventually extend our research on the ‘moko’ drum names to other areas in Alor (e.g., the Kabola and Kamang territories) and in Timor. In particular, the Kabola context looks promising, also considering the partial list of drum denominations available at the Kalabahi Museum in Alor Regency (we did not include it in our data set, because we have not been able to verify it with a sufficient number of local native speakers yet).

## Ethics Statement

Informed consent was obtained from all speakers and consultants involved in the study (for in-person interviews, online interviews, data collection, personal data protection and publication, data management and publication in general, and individual compensation) and all ethical and technical aspects have been represented and listed in the informed consent forms.

## CRediT authorship contribution statement

**Shiyue Wu:** Conceptualization, Methodology, Data curation, Writing – original draft, Writing – review & editing. **Francesco Perono Cacciafoco:** Supervision, Methodology, Data curation, Writing – original draft, Writing – review & editing.

## Data Availability

Name List of `Moko' Drums in Abui, Sawila, and Kula (Original data) (Mendeley Data).
